# Nursing Progress and Its Developments: XI. The Grandeur of the Nation's Nursing Services

**Published:** 1918-03-30

**Authors:** 


### THE MATRONS' AND SISTERS' DEPARTMENT.

### NURSING PROGRESS AND ITS DEVELOPMENTS.

### XI.—The Grandeur of the Nation's Nursing Services.

How few among the women who make up the Nursing Service are conscious of the great temple in which they are living stones! Collectively they make part of a body more deeply touched with the spirit of sacrifice, more skilfully trained for the performance of duty, more generally imbued with high ideals than any other body of workers. Yet individually each member of the profession tends to have her attention distracted from this aspect of the nursing community. In her outlook over the profession she is apt to limit herself to an estimate of her colleagues, yields to the subtle temptation to depreciate another's aims and character, and so misses the gladness of finding herself one of a corporation the like of which the world has never yet seen.

It is not too much to say that the nation owes its salvation in this war in equal degree to the Medical and Nursing Services which have stood behind its fighting men and have enabled them to persevere under the incredible hardships and sufferings of modern warfare. Nothing is more astonishing in the evolution of trained nursing than that this magnificent body of women has still no corporate existence, no recognised rights, no officers to enforce its claims with authority, no proper code of admission to its privileges and responsibilities, no organised system of defence when attacked, no adequate provision for such as encounter disaster in the course of duty. Its members have not even a name which can distinguish them from the lower ranks of domestic workers. Their distinctive uniform may be copied by thieves and outcasts and used to the basest ends. Any ignorant person who pleases may call herself a nurse, receive payment as a nurse, and bring scandal on the nursing profession by malpraxis or crime.

Is it not difficult to believe that so far on in the twentieth century, in the land which gave birth to trained nursing, these things should be true?

What is wanted throughout the length and breadth of the Nursing Service is a vivid perception of the perils which lie in disunion. Nurses must throw to the winds the old selfish "What is the good of it?" cry. They need no mere debating society to take up their interests, no elaborate network of by-laws and regulations for ever treading water and making no progress. Clubs and benefit societies are very well in their way. But the Nursing Service calls for something infinitely higher and more powerful. It must build for itself a symbol of union, founded on the adhesion of every trained nurse in the kingdom, governed in the interests of its members by women whom they themselves have chosen on the ground of their proven fitness to construct a living policy. The College of Nursing now rising out of the mist already gives promise of fulfilling these requirements. There must be no oblivion of the high purposes it has set out to achieve. To lose sight amid the multiplicity of detail of its national aims would cramp and stifle it from the beginning. It must ever be borne in mind that every association of persons for a common object ultimately becomes what its members will it to become. If they lose sight of the whole and think merely of narrow-bread-and-cheese interests, if they wrangle and intrigue against each other, thinking more of individual claims to notoriety than of the common aims, then the structure will be petty, its influence slight. But if the ideals towards which the profession is moving be kept ever in sight, if the good of the whole be more potent with those who build than the rounding of particular corners, then all the rest will follow. Imagination must guide and beauty of conception will carry with it the redress of all grievances. There is more practical wisdom in this course than is generally understood. What really raises the pay of workers is not so much agitation for higher fees, nor even combination with this limited aim in view. It is the lifting of the entire profession so that every member of it is worth more. That is the direction in which the Nursing Service, even without a central body, and seriously handicapped as it has ever been by this want, has constantly moved.

The women who realise the grandeur of their own calling are the women who are capable of inspiring high ideals for the College and of bringing them into being. When once nurses realise that they are members of a noble confraternity, they will not rest till they have a corporate existence worthy of their work and destiny.

